# Effects of Optimized Acupuncture and Moxibustion Treatment on Depressive Symptoms and Executive Functions in Patients With Post-Stroke Depression: Study Protocol for a Randomized Controlled Trial

**DOI:** 10.3389/fneur.2022.833696

**Published:** 2022-03-18

**Authors:** Meng Luo, Zhaoyuan Duan, Xiaolei Song, Chengmei Liu, Ruiqing Li, Kaiqi Su, Yanjie Bai, Xiaodan Wang, Wenbin Fu, Jing Gao, Xiaodong Feng

**Affiliations:** ^1^Department of Rehabilitation Medicine, Henan University of Chinese Medicine, Zhengzhou, China; ^2^Rehabilitation Center, The First Affiliated Hospital of Henan University of Chinese Medicine, Zhengzhou, China; ^3^The Second Clinical College, Guangzhou University of Chinese Medicine, Guangzhou, China; ^4^Department of Acupuncture and Moxibustion, The Second Affiliated Hospital of Guangzhou University of Chinese Medicine (Guangdong Provincial Hospital of Chinese Medicine), Guangzhou, China

**Keywords:** post-stroke depression (PSD), acupuncture, moxibustion, auricular intradermal acupuncture, executive function, fMRI, randomized controlled trial, protocol

## Abstract

**Background:**

Post-stroke depression (PSD), a common neuropsychiatric comorbidity after stroke, has a negative impact on the functional recovery and quality of life of survivors. It lacks effective therapeutic drugs with good curative effects and few adverse reactions. Preliminary experiments have shown that the optimized acupuncture and moxibustion treatment (OAMT), including acupuncture, moxibustion, and auricular intradermal acupuncture, improved depressive symptoms and neurological deficits in patients with PSD. However, the evidence for its effectiveness is still insufficient. Hence, we designed this study to evaluate the efficacy and safety of the OAMT in the treatment of PSD and to explore its possible mechanism from the perspective of executive functions.

**Methods/Design:**

This is a randomized controlled trial, which comprises a total of 134 patients with PSD. Participants are randomized into intervention group and control group at a 1:1 ratio. All treatments are given five times per week for 4 weeks. The primary outcome is the severity of depression, which is evaluated by the Hamilton Depression Scale-17 (HAMD-17) and the Beck Depression Rating Scale (BDI). Secondary outcomes are executive abilities, which are measured by several neuropsychological tests, including the Stroop Color and Word Test (SCWT), the Trial Making Test (TMT), the Digit Symbol Substitution Test (DSST), and the Matrix Reasoning Test (MRT). All outcomes have been evaluated at baseline and weeks 4, 8, 12, and 20. At the same time, functional MRI (fMRI) is used to measure the functional connectivity in the cognitive control network (CCN) at baseline and 4 weeks after intervention.

**Discussion:**

This study aims to provide high-quality evidence for the efficacy and safety of the OAMT for treating PSD. In addition, this trial is the first trial to explore if the improvement condition of depression in the OAMT group is related to the improvement of executive functions and the favorable changes in the structure.

**Clinical Trial Registration:**

Chinese Clinical Trial Registry, identifier: ChiCTR2100048431.

## Introduction

Stroke is the leading cause of death and disability worldwide, contributing to a high burden of disease (1). Post-stroke depression (PSD) is a common neuropsychiatric comorbidity after stroke. It is reported that 20–65% of people suffer from PSD, and the cumulative percentage of patients with depression in the first 5 years after stroke is 39–52% (2, 3). In addition, PSD is not only related to the poor results of rehabilitation but also increases the risk of stroke recurrence and mortality. Unluckily, most guidelines for stroke do not address the best way to identify and treat depression in these patients, and existing studies on PSD are still insufficient (4–8).

A Cochrane review showed that there is limited evidence on the effectiveness of drug interventions for PSD (9). Commonly used drugs, such as serotonin reuptake inhibitors (SSRIs) and serotonin norepinephrine reuptake inhibitors (SNRIs), however, may cause a series of adverse reactions, as well as bring risks, including potential cerebral hemorrhage, myocardial infarction, and all-cause mortality (10–12). Therefore, it is of great significance to seek effective complementary and alternative therapies with a few side effects.

Acupuncture has a history of more than 2,000 years in China and is used to treat neuropsychiatric diseases such as stroke and depression. A meta-analysis shows that acupuncture significantly reduces the degree of PSD and has a better safety profile than antidepressants (13). Its antidepressant effect is reflected in the improvement of the Hamilton Depression Scale-17 (HAMD-17) score and the quality of life (14–18). Compared with the routine single acupuncture treatment, the addition of auricular intradermal acupuncture and moxibustion could consolidate and prolong the curative effect (19, 20). However, the efficacy of the optimized acupuncture and moxibustion treatment (OAMT) in the treatment of patients with PSD remains to be proven, and its mechanism has not been fully elucidated.

Depressive symptoms after stroke have been reported to be closely related to executive dysfunction (21). Pohjasvaara et al. (22) showed that executive dysfunction was detected in 40.6% (*n* = 104) of 256 patients 3–4 months after stroke, and this dysfunction was proven to be related to depressive symptoms. In the same vein, patients with PSD with executive dysfunction show more severe depressive symptoms and have a significantly higher incidence of cerebral infarction in the frontal lobe-subcortical circuit compared with patients with PSD without executive dysfunction (21). In neuroanatomy, links between the two have also been reported (23). Both are associated with alterations in intrinsic and extrinsic structural and functional connectivity in the convolutional neural network (CNN). Specifically, both may have structural disconnections in frontal, parietal, and subcortical areas (24–26), and these disconnections have contacts with lower intrinsic functional connectivity in the CNN (27). Cognitive control network (CCN) is the frontal parietal loop (28), which participates in top-down, attention-dependent executive functions such as decision-making and task switching (29–31). The dorsolateral prefrontal cortex (DLPFC) is an important node of this network (32), and it has been proven that the left DLPFC functional connectivity is negatively correlated with the severity of PSD (33). Several studies using DLPFC as a seed have reported the decreased functional connectivity within the CCN after depression (34–36). Alexopoulos et al. (35) pointed out that lower CCN connectivity can predict the lower recovery rate and symptom improvement of depressed individuals after taking escitalopram. Ye et al. (36) found that the node centrality of DLPFC in patients with depression is lower than that of normal people, which also reflects the weakening of the network function of CCN in patients with depression. Therefore, DLPFC is used as a seed to observe the functional connectivity in the CCN, which may be a key for exploring the neural mechanism of the OAMT in the treatment of patients with PSD. In addition, studies have shown that acupuncture is conducive to relieving executive dysfunction (37, 38), which may be resulted from taking an effect on the central nervous system through local reflex, affecting neurotransmitter levels, etc., to regulate the executive control system (39, 40). Therefore, this trial not only evaluates the degree of depression and executive functions in patients with PSD but also observes the CCN network's functional connectivity, providing stronger evidence for the efficacy and mechanism of the OAMT from multiple perspectives.

## Methods and Analysis

### Design and Setting

**Figure 1:**
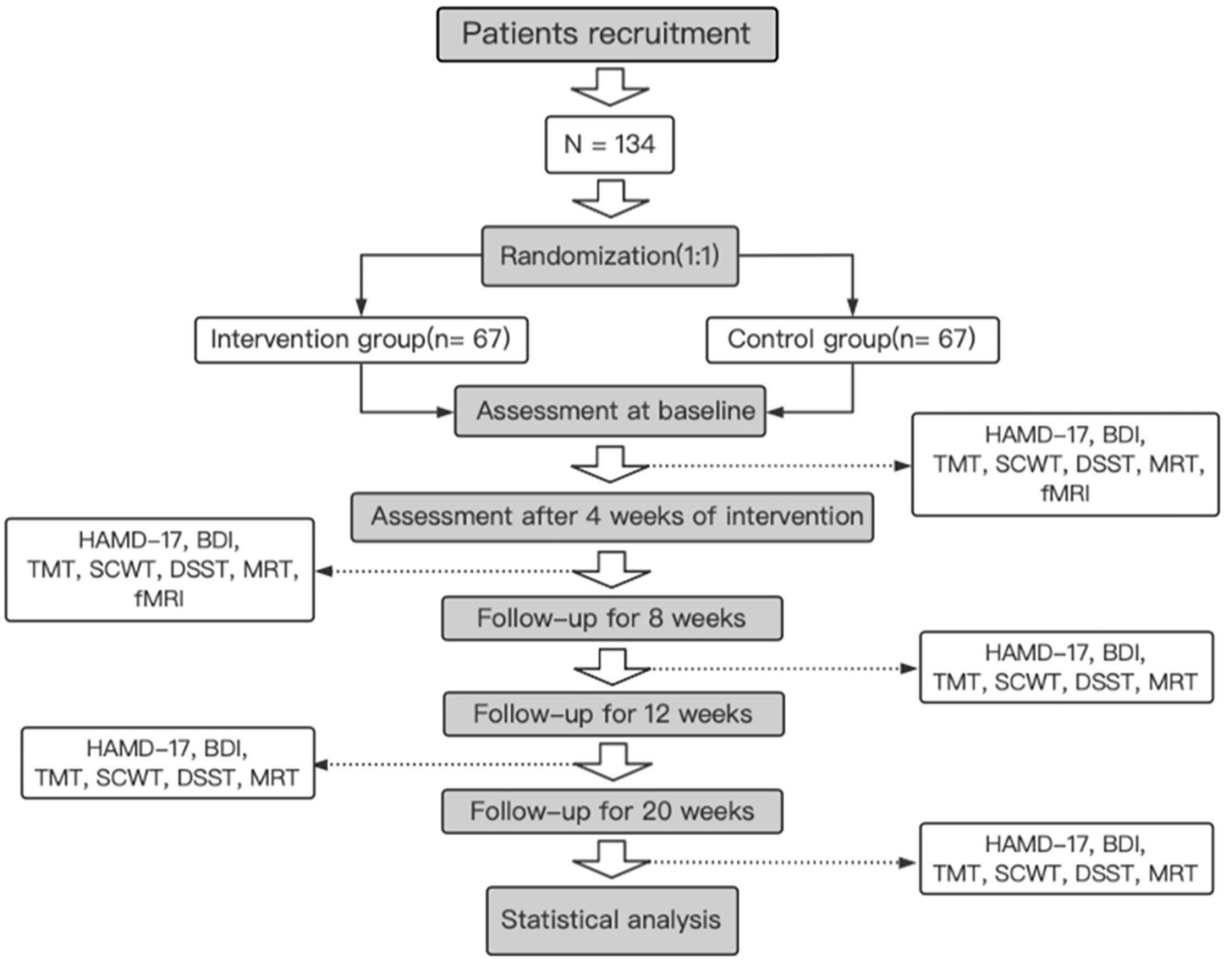
A flow chart of the trial.

This is a prospective, randomized controlled trial conducted by the First Affiliated Hospital of Henan University of Chinese Medicine. A total of 134 patients who meet the inclusion and exclusion criteria are randomly divided into two groups. One group receives the OAMT and routine medicine and rehabilitation treatment, and another group receives routine medicine and rehabilitation treatment only. All treatments are provided five times per week for 4 weeks. The primary outcome is the severity of depression, which is evaluated by the HAMD-17 and the Beck Depression Rating Scale (BDI). Secondary outcomes are executive abilities, which are measured by several neuropsychological tests, including the Stroop Color and Word Test (SCWT), Trial Making Test (TMT), Digit Symbol Substitution Test (DSST), and Matrix Reasoning Test (MRT). All outcomes are evaluated at baseline and weeks 4, 8, 12, and 20. At the same time, functional MRI (fMRI) is used to measure the functional connectivity in the CCN at baseline and 4 weeks after intervention. The study's flow chart is shown in Figure 1, and the process chart is shown in Table 1.

**Table 1:** A process chart of the trial.

	**Study period**					
**Timepoint**	**Enrollment**	**Baseline**	**Treatment phase**	**Follow-up phase**		
	**−1 week**	**0 week**	**4 weeks**	**8 weeks**	**12 weeks**	**20 weeks**
**Enrollment**						
Eligibility screen	×					
Informed consent	×					
Medical history	×					
Merger disease	×					
Randomization		×				
**Interventions**						
Intervention group		×	×			
Control group		×	×			
**Assessments**						
HAMD-17		×	×	×	×	×
BDI		×	×	×	×	×
SCWT		×	×	×	×	×
TMT		×	×	×	×	×
DSST		×	×	×	×	×
MRT		×	×	×	×	×
fMRI		×	×			
Safety evaluation		×	×			
Adverse events		×	×	×	×	×

### Recruitment of Participants

This trial is conducted at the First Affiliated Hospital of Henan University of Chinese Medicine, China. Patients who meet the criteria are recruited through the outpatient and inpatient systems and advertisements. The recruitment begins on January 1, 2022 and is expected to end in June 2023.

### Inclusion Criteria

Participants who meet the following criteria are included

(1) Aged between 40 and 85.
(2) Diagnosed with ischemic stroke, and met the diagnostic criteria for “Depressive disorder due to another medical condition” in the Diagnostic and Statistical Manual of Mental Disorders, Fifth Edition (DSM-V) (41).
(3) Had a first episode of stroke within 6 months.
(4) The HAMD-17 score >7 points, or <24 points.
(5) Have not taken antidepressants systematically.
(6) Patients or their immediate families sign an informed consent and voluntarily participate in this study.

### Exclusion Criteria

Participants with the following conditions are excluded:

(1) The HAMD-17 score ≤ 7 points, or ≥24 points.
(2) Patients with obvious suicidal tendencies assessed by specialists.
(3) Diagnosed with depression, cognitive impairment, schizophrenia, bipolar disorder, substance abuse, or other mental disorders before stroke.
(4) Patients who are taking antidepressant drugs.
(5) Patients who have severe heart, liver, kidney, and other medical diseases or tumors.
(6) Patients who are diagnosed with bleeding disorders, coagulation dysfunction, and skin infections are not suitable for acupuncture and moxibustion treatment.
(7) Pregnant or lactating women.
(8) Contraindication for an MRI examination.
(9) Patients participating in any other clinical trials.

### Randomization

The randomization sequence is generated by an independent statistician from the Henan Evidence-based Medicine Center of Traditional Chinese Medicine, using the PROCPLAN process of the SAS statistical analysis system. Then, the randomization sequence is placed in opaque sealed envelopes and assigned to the eligible patients who can be included.

### Blinding

Due to the characteristics of the OAMT and the technical limitations, we are unable to conduct a double-blind study design. However, evaluators and statisticians of the outcome are blinded to the assignments. Patients are treated separately to avoid communication, and the OAMT sessions are strictly performed by licensed and experienced acupuncturists. Acupuncturists provide any information about the allocation to the patients, evaluators, or statisticians. In the process of data management and statistical analysis, a professional statistician who is not in this study is invited to undertake analysis tasks.

### Interventions

Patients receive treatments in separate rooms five times a week for 4 weeks. All treatments are performed by licensed acupuncturists with more than 3 years of experience in practice. The location of the acupoints follows the WHO standards (42–44). At the same time, routine medicine and rehabilitation treatment of each patient is performed by physicians and therapists who do not know the allocation. The interventions of the two groups are as follows.

### Intervention Group

Patients receive the OAMT and routine medicine and rehabilitation treatment once a day, five times a week, and for 4 weeks. The OAMT consists of acupuncture, moxibustion, and auricular intradermal acupuncture. The specific operations are as follows:

#### Acupuncture

After skin disinfection with 75% alcohol cotton swabs, patients receive acupuncture at Baihui (GV20), Shenting (GV24), Yintang (GV29), bilateral Hegu (LI4), Jiuwei (CV15), Zhongwan (CV12), Qihai (CV6), bilateral Sanyinjiao (SP6), and bilateral Taichong (LR3). Disposable sterile needles (0.25 ×25 mm; Huatuo, Suzhou Medical Appliance Fact. 215005 Suzhou, China) are used. Using the tube-guide method, the needles are inserted into the acupoints and operated for the sense of “De qi.” Specific acupuncture methods of each acupoint are shown in Table 2. The needles are kept in the acupoints for 30 min.

**Table 2:** Specific acupuncture methods of each acupoint.

**Acupoints**	**Location**	**Insert angle**	**Insert depth**
Baihui (GV20)	On the head, 5 cun directly above the midpoint of the anterior hairline	15°	0.5 cun
Shenting (GV24)	On the head, 0.5 cun directly above the midpoint of the anterior hairline	15°	0.5 cun
Yintang (GV29)	On the head, at the intersection of the line between the two brows and the front midline	15°	0.5 cun
Hegu (LI4) (bilateral)	On the dorsum of the hand, between the first and second metacarpal bones, approximately in the middle of the second metacarpal bone on the radial side	90°	0.5 cun
Jiuwei (CV15)	On the anterior median line of the upper abdomen, 1 cun below the Xiphisternal Synchondrosis	45°	0.5 cun
Zhongwan (CV12)	On the anterior median line of the upper abdomen, 4 cun above the navel	90°	1 cun
Qihai (CV6)	On the lower abdomen, on the front midline, 1.5 cun below the navel	90°	0.5 cun
Sanyinjiao (SP6) (bilateral)	On the medial side of the shank, 3 cun above the medial malleolus, by the posterior of the tibia	90°	1 cun
Taichong (LR3) (bilateral)	On the dorsum of the foot, in the depression proximal to the first metatarsal space	45°	0.5 cun

*A 1 cun (≈20 mm) is defined as the width of the interphalangeal joint of the patient's thumb*.

#### Moxibustion

There are two acupoint selection plans used alternatively: (1) Feishu (BL13), Geshu (BL17), Danshu (BL19), and Yongquan (KI1). (2) Pohu (BL42), Geguan (BL46), Yanggang (BL48), and Yongquan (KI1). The location of the acupoints is shown in Table 3. The ignited moxa roll (herbal preparation of Artemisia vulgaris, Z32021062, Oriental Moxa Co., Suzhou, China) is applied 3 cm above the skin of acupoints, making the patient feel warm. When the skin turns red, the burning ash is moved away in time to avoid burning injury. Moxibustion for 30 min each time.

**Table 3:** The location of moxibustion acupoints.

**Acupoints**	**Location**
Feishu (BL13)	Under the spinous process of the third thoracic vertebrae, at the midpoint of the line between the medial edge of the scapula and the spine
Geshu (BL17)	Under the spinous process of the seventh thoracic vertebrae, at the midpoint of the line between the medial edge of the scapula and the spine
Danshu (BL19)	Under the spinous process of the ninth thoracic vertebrae, at the midpoint of the line between the medial edge of the scapula and the spine
Yongquan (KI1)	On the mid-line of the sole of the foot, 2/3 of the way forward from the back of the heel
Pohu (BL42)	Under the spinous process of the third thoracic vertebrae, at the medial edge of the scapula
Geguan (BL46)	Under the spinous process of the seventh thoracic vertebrae, at the medial edge of the scapula
Yanggang (BL48)	Under the spinous process of the second lumbar vertebra, 1 cun beside the spine

*A 1 cun (≈20 mm) is defined as the width of the interphalangeal joint of the patient's thumb*.

#### Auricular Intradermal Acupuncture

**Figure 2:**
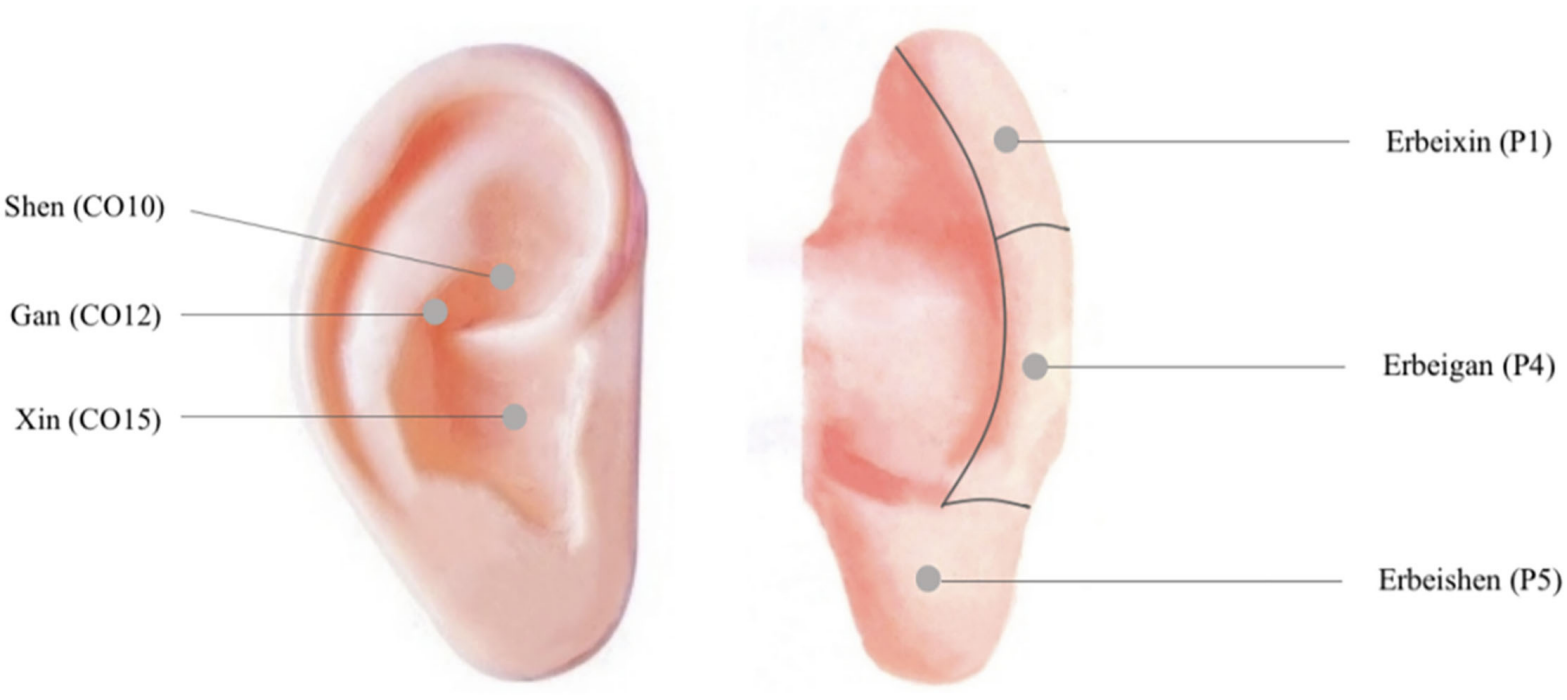
The location of auricular intradermal acupuncture acupoints.

Following the acupuncture and moxibustion treatment, patients receive auricular intradermal acupuncture. There are two acupoint selection plans used alternatively: (1) Xin (CO15), Gan (CO12), and Shen (CO10) and (2) Erbeixin (P1), Erbeigan (P4), and Erbeishen (P5). The location of the acupoints is shown in Figure 2. After skin disinfection, the acupuncturist holds a sterile intradermal needle (0.22 × 1.5 mm, ZHONGYANTAIHE, AN2016, Wujiang Shenling Medical Equipment Co., Wujiang, China) to penetrate the auricle's skin. The insert angle is < 10°. Then, the needle is fixed on the acupoint with a medical tape. Intradermal needles are kept for 4 h in each session.

### Control Group

Patients receive routine medicine and rehabilitation treatment for 4 weeks. For stroke and underlying diseases (such as hypertension and diabetes), we have provided symptomatic treatments, including antiplatelet aggregation, lowering lipids and stabilizing plaques, blood pressure, and blood sugar treatment, and have tried to avoid the drugs that affect mental factors, referring to the Guidelines for the Prevention and Treatment of Cerebrovascular Diseases in China (45). At the same time, routine rehabilitation treatment, such as exercise therapy and physical therapy, is provided equally.

### Sample Size

The main outcome of this study is the improvement of depressive symptoms. Our team's preliminary pre-experiment (*n* = 30) showed that the OAMT for 4 weeks can reduce the HAMD-17 score by 14.53 ± 1.66. When α = 0.05, β = 0.1, 1 – β = 0.9, and the sample size of the intervention group and the control group are equal, the normal distribution quantile table shows that *Z*_α/2_ = 1.96, *Z*_β/2_ = 1.282, σ = 1.66 means the SD of the intervention group and δ = 1.28 means the mean difference of the intervention group. Bring the data in the following formula:


$$\begin{array}{l} \text{N }=\;2\;\times \;{\left[\left({\text{Z}}_{\text{α}}/2+{{\text{Z}}_{\text{β}}}_{/2}\right)\;\times \sigma /\delta \right]}^{2} \end{array}$$

After the calculation, the sample size is N = 60. Taking the 10% dropout rate into account, the sample size is 67 patients per group (134 in total). All 134 participants receive fMRI scans, and this sample size is much larger than the 12 cases per group required by the technical requirements of quality control and network analysis of acupuncture brain functional imaging (46), which can make the test results more reliable.

### Outcome Measures

Participants are evaluated at baseline and weeks 4, 8, 12, and 20. All evaluations are conducted by researchers who are blinded to the treatment allocation.

#### Primary Outcomes

Primary outcomes are the HAMD-17 and the BDI.

##### Hamilton Depression Scale-17

The HAMD-17 is one of the most widely used scales in the evaluation of depression and is used to assess the severity of depression (47, 48). It consists of 17 items. The higher the score, the more severe the depression (49). The classification is as follows: normal (< 7), possible depression (7–17), diagnosed depression (18–24) and severe depression (> 24).

##### Beck Depression Rating Scale

The BDI is a commonly used self-evaluation scale to measure depression. It measures the intensity of depression by judging the main symptoms of depression syndrome (50). The advantages of the BDI lie in its international spread, high internal consistency of psychiatric and non-psychiatric samples, high content validity, high sensitivity to changes, and high convergence validity. The entire scale includes 21 groups of items, each group has four options, and each option corresponds to a certain score. A total score of <10 means no depression. The higher the score, the more severe the depression (51).

#### Secondary Outcomes

Secondary outcomes will be obtained using several neuropsychological tests and fMRI scanning. Neuropsychological tests include SCWT, TMT, DSST, and MRT.

##### Stroop Color and Word Test

The SCWT is a neuropsychological test widely used for experimental and clinical purposes. It can measure not only the ability to suppress cognitive interference but also a variety of cognitive functions (such as attention, processing speed, cognitive flexibility, and working memory) (52). The test is generally divided into three parts: (1) quickly name the color pictures; (2) quickly read the nouns that represent the name of the color; and (3) a set of cards have been presented with the nouns representing the color names written in colors different from the meaning of the words. A researcher checks the patient's ability to distinguish color names from actual colors and quickly read nouns representing color names. The degree to which the patient is affected by the color of the words is used as an index to measure his/her cognitive control ability.

##### Trial Making Test

The TMT is a commonly used neuropsychological test (53), and its reliability and effectiveness have been previously proven (54). TMT consists of two parts, involving visual search and scanning capabilities, processing speed, mental flexibility, and executive functions. TMT-A requires a patient to connect the circled numbers distributed on a piece of paper one by one. The requirements of TMT-B are similar to those of TMT-A, but the patient must alternate between numbers and graphics. The total score is calculated based on the task completion time and the accuracy rate.

##### Digit Symbol Substitution Test

The DSST was originated from the Wechsler Adult Intelligence Scale (55), which can evaluate participants' abilities related to digital decoding, memory, attention, and operating speed (56). Patients are provided with a table showing various symbols and matching numbers, and they are asked to write down matching symbols for each number in several rows of numbers. The score is the number of correctly coded numbers completed in 120 s (57).

##### Matrix Reasoning Test

The MRT is a sub-test of the Wechsler Intelligence Abbreviation Scale (58, 59), which measures non-verbal reasoning ability. It is composed of a sequence or a set of graphic matrixes. Patients are required to select the patterns or symbols that can be filled in the vacant part according to the pattern or symbol change rules in the incomplete sequence or graphic matrix. The score is the number of items completed correctly.

### Functional MRI

An Ingenia 3.0 Tesla MRI scanner (Philips MedicalSystems, Best, Netherlands) with a head orthogonal coil is used for fMRI data acquisition. Participants are instructed to lie supine, close their eyes, and keep quiet and awake. High-resolution three-dimensional T1-weighted MRIs are collected at the beginning of the scanning session with the following parameters: TR = 1,900 ms, TE = 2.56 ms, flip angle = 9°, FOV = 250 × 250 mm, matrix size = 246 × 246 mm, and slice thickness = 1 mm. Then, the resting-state data are acquired as follows: TR = 2,000 ms, TE = 30 ms, flip angle = 90°, FOV = 240 × 240 mm, matrix size = 64 × 64 mm, slice thickness = 5 mm, and slice number = 32 slices.

### Safety Evaluation and Adverse Events

Safety indicators are tested before and after the treatment, including general physical examination (blood pressure, pulse, and breathing), ECG, blood routine, urine routine, stool routine, and liver and kidney function tests. In the case report forms (CRFs), adverse events such as pain, fainting, local infection, and allergies are recorded in detail. Serious adverse events, such as death or life-threatening events, are reported to the researcher immediately and reported to the ethics committee within 24 h. In those circumstances, the research team gives the patient treatment and suggestions based on the situation, evaluates whether he/she continues to participate in the research, and compensates him/her accordingly.

### Data Management

The trained evaluators use CRFs to record patients' information and data in detail and import them into an electronic database. After the study is completed, the paper CRFs are stored in a locked cabinet. Meanwhile, the electronic database has also been locked, so researchers do not able to modify the data. Participants' personal data are kept anonymously and strictly confidential. For patients who discontinue or leave the trial, we obtain their data by telephone with their consent. The Data Monitoring Committee of the Rehabilitation Center of the First Affiliated Hospital of Henan University of Chinese Medicine is established. They are independent of researchers to monitor the trial progress, regularly monitor the safety of the trial, and check the completeness and accuracy of the CRFs.

### Data Analysis

#### Clinical Data Analysis

We invite third-party professional statisticians who do not know the trial protocol to conduct a statistical analysis and participate in the whole process comprising trial design, implementation, and data analysis. If the necessary data are available, subgroup analyses are performed by different clinicopathological features, such as gender, age, time of stroke onset, and stroke severity. A statistical analysis is performed using SPSS 22.0.

The data are statistically described by mean ± SD. Continuous variables are compared using Student *t*-test or Wilcoxon rank sum test, and categorical variables are compared using Pearson χ^2^ test or Wilcoxon rank-sum test. The comparison between the groups is carried out by an independent sample *t*-test or the Mann–Whitney *U*-rank-sum test based on whether the measurement data present a normal distribution and homogeneous variance. When doing a statistical analysis with two-tailed testing, the significance level is set at 5%. When *p* < 0.05, differences have been considered as statistically significant.

#### MRI Data Analysis

The SPM8 software platform (SPM8, Wellcome Department of Imaging Neuroscience, London, UK; http://www.fil.ion.ucl.ac.uk/spm/) is used to preprocess the MRI data. Referring to the study of DLPFC resting-state connectivity of patients with PSD (33), we select the seeds from bilateral DLPFC, draw the region of interest (ROI) in WFU Pickatlas (60) to tap into the CCN, and perform a whole-brain regression analysis. The DLPFC resting-state connectivity in the CCN is compared between the intervention group and the control group. A paired *t*-test is used to evaluate brain changes in each group through an intragroup analysis (before and after the treatment). We include factors such as age and gender as covariates in the data analysis. The Pearson correlation coefficient is used to analyze the relationship between the improvement value of the correlation scales and the change of fMRI image data.

### Quality Control

Qualified acupuncturists, physicians, therapists, and statisticians in the First Affiliated Hospital of Henan University of Chinese Medicine monitored and revised this trial protocol. All relevant personnel are trained in accordance with the prepared standard operating procedure (SOP) to ensure consistency in the comprehension and implementation of interventions and evaluations. At the same time, a quality control team is established to conduct quality control, and the qualified clinical trial experts are invited to supervise the trial once a month.

## Discussion

There is a large volume of published studies showing that acupuncture and related therapies effectively improved depression, and the combination therapy appeared to have superior efficacy (14–16, 19, 20). However, these studies used diverse acupoint selection plans and intervention methods, and lacked a unified standard acupuncture program. Moreover, they remain narrow in focus dealing only with efficacy without exploring the mechanism. The OAMT is a combination therapy based on the acupuncture theory, our previous experiments, and practical experience. It was developed according to the theory of “ShuGanTiaoShen” (smoothing the liver and regulating mental activities). Chinese doctor believes that the cause of PSD is closely related to “Liver Failing to Maintain Normal Flow of Qi” and “Disorder of Cerebral Soul” (61, 62). Referring to this, this study select acupoint plans with the effect of “ShuGanTiaoShen.”

Based on the clinical practice and previous experimental results of the First Affiliated Hospital of Henan University of Chinese Medicine, we completed the first draft of the protocol for this study. Subsequently, the ethics committee of the hospital discussed and revised the draft, and the final version of the protocol was unanimously approved by the ethics committee.

This trial is the first trial to explore the OAMT treatment of the PSD population from the executive control system. It has been proven that the reduction of functional connectivity in the CCN affects depression vulnerability and maintenance through an impact on the cognitive control of emotional information (63, 64). In other words, neurological deficits are considered as a basis for the difficulty of cognitive control mechanisms (e.g., attention control, inhibition, and reassessment) that support emotion regulation. These deficits may hinder the function of CNN. Therefore, observing neurological defects of CCN through fMRI is of certain significance to explore the mechanism of the OAMT in the treatment of PSD.

However, this study has some limitations. First, it is a single-center study in which patients from different areas cannot be recruited. Second, although this study followed the principle of separation of researchers, operators, and statisticians due to the nature of acupuncture and moxibustion treatment, we did not use blind methods in the OAMT. This may cause partial deviations in the results. Despite all of these, we strive to standardize the process of this study to provide high-quality medical evidence for the OAMT, as well as an optimized plan as a complementary therapy for PSD.

## Ethics Statement

The studies involving human participants were reviewed and approved by the Ethics Committee of the First Affiliated Hospital of Henan University of Chinese Medicine (reference number: 2021HL-184-01). The patients/participants provided their written informed consent to participate in this study.

## Author Contributions

XF and JG designed this study. ML and ZD drafted the manuscript together. XS revised this manuscript and conducted the preliminary pre-experiment. CL, RL, KS, and XW recruited patients and provided treatment. WF and YB conducted a statistical analysis in the pre-experiment. All authors agreed to the final version of this manuscript.

## Funding

This study was supported by the National Natural Science Foundation of China (Grant Nos. 82104973 and 81574042), the Ministry of Science and Technology of the People's Republic of China (Grant No. 2018YFC1706004), the Department of Science and Technology of Henan Province (Grant No. 222102310715), and the Henan Provincial Administration of Traditional Chinese Medicine (Grant No. 2022ZY1162).

## Conflict of Interest

The authors declare that the research was conducted in the absence of any commercial or financial relationships that could be construed as a potential conflict of interest.

## Publisher's Note

All claims expressed in this article are solely those of the authors and do not necessarily represent those of their affiliated organizations, or those of the publisher, the editors and the reviewers. Any product that may be evaluated in this article, or claim that may be made by its manufacturer, is not guaranteed or endorsed by the publisher.

## Abbreviations

PSD: post-stroke depression
OAMT: optimized acupuncture and moxibustion treatment
HAMD-17: Hamilton Depression Scale-17
BDI: Beck Depression Rating Scale
TMT: Trial Making Test
SCWT: Stroop Color and Word Test
DSST: Digit Symbol Substitution Test
MRT: Matrix Reasoning Test
CCN: cognitive control network
SSRIs: serotonin reuptake inhibitors
SNRIs: serotonin norepinephrine reuptake inhibitors
DLPFC: dorsolateral prefrontal cortex
DSM-V, Diagnostic and Statistical Manual of Mental Disorders: Fifth Edition
SOP: standard operating procedure
CRFs: case report forms.

## References

[1] Collaborators GBDS. Global, regional, and national burden of stroke, 1990-2016: a systematic analysis for the Global Burden of Disease Study 2016. Lancet Neurol. (2019) 18:439–58. 10.1016/S1474-4422(19)30034-1 30871944 PMC6494974

[2] Sharma GS Gupta A Khanna M Prakash NB. Post-stroke depression and its effect on functional outcomes during inpatient rehabilitation. J Neurosci Rural Pract. (2021) 12:543–9. 10.1055/s-0041-1731958 34295110 PMC8289524

[3] Ayerbe L Ayis S Wolfe CD Rudd AG. Natural history, predictors and outcomes of depression after stroke: systematic review and meta-analysis. Br J Psychiatry. (2013) 202:14–21. 10.1192/bjp.bp.111.107664 23284148

[4] Carod-Artal FJ. Post-stroke depression (I). Epidemiology, diagnostic criteria and risk factors. Rev Neurol. (2006) 42:169–75. 10.33588/rn.4203.2005049 16475139

[5] Dafer RM Rao M Shareef A Sharma A. Poststroke depression. Top Stroke Rehabil. (2008) 15:13–21. 10.1310/tsr1501-13 18250069

[6] Sturm JW Donnan GA Dewey HM Macdonell RA Gilligan AK Srikanth V . Quality of life after stroke: the North East Melbourne Stroke Incidence Study (NEMESIS). Stroke. (2004) 35:2340–5. 10.1161/01.STR.0000141977.18520.3b 15331799

[7] Morris PL Robinson RG Andrzejewski P Samuels J Price TR. Association of depression with 10-year poststroke mortality. Am J Psychiatry. (1993) 150:124–9. 10.1176/ajp.150.1.124 8417554

[8] Morris PL Robinson RG Samuels J. Depression, introversion and mortality following stroke. Aust N Z J Psychiatry. (1993) 27:443–9. 10.3109/00048679309075801 8250788

[9] Hackett ML Anderson CS House A Xia J. Interventions for treating depression after stroke. Cochr Database Syst Rev. (2008) CD003437. 10.1002/14651858.CD003437.pub3 18843644

[10] Coupland C Dhiman P Morriss R Arthur A Barton G Hippisley-Cox J. Antidepressant use and risk of adverse outcomes in older people: population based cohort study. BMJ. (2011) 343:d4551. 10.1136/bmj.d4551 21810886 PMC3149102

[11] Smoller JW Allison M Cochrane BB Curb JD Perlis RH Robinson JG . Antidepressant use and risk of incident cardiovascular morbidity and mortality among postmenopausal women in the Women's Health Initiative study. Arch Intern Med. (2009) 169:2128–39. 10.1001/archinternmed.2009.436 20008698 PMC10204121

[12] Hackam DG Mrkobrada M. Selective serotonin reuptake inhibitors and brain hemorrhage: a meta-analysis. Neurology. (2012) 79:1862–5. 10.1212/WNL.0b013e318271f848 23077009

[13] Liu R Zhang K Tong QY Cui GW Ma W Shen WD. Acupuncture for post-stroke depression: a systematic review and meta-analysis. BMC Comp Med Ther. (2021) 21:109. 10.1186/s12906-021-03277-3 33794857 PMC8017746

[14] Chen SX Liu FF. [Effect of tiaoshen kaiyu acupuncture (regulating vitality and dredging stasis) combined with psychological intervention on patients of mild depression after stroke]. Zhen Ci Yan Jiu. (2018) 43:39–43. 10.13702/j.1000-0607.170049 29383893

[15] You Y Zhang T Shu S Qian X Zhou S Yao F. Wrist-ankle acupuncture and Fluoxetine in the treatment of post-stroke depression: a randomized controlled clinical trial. J Tradit Chin Med. (2020) 40:455–60. 10.19852/j.cnki.jtcm.2020.03.014 32506860

[16] Nie RR Huang CH. [Post-stroke depression treated with acupuncture and moxibustion: an evaluation of therapeutic effect and safety]. Zhongguo Zhen Jiu. (2013) 33:490–4. 23967632

[17] Zhang GJ Shi ZY Liu S Gong SH Liu JQ Liu JS. Clinical observation on treatment of depression by electro-acupuncture combined with Paroxetine. Chin J Integr Med. (2007) 13:228–30. 10.1007/s11655-007-0228-0 17898957

[18] Smith CA Armour M Lee MS Wang LQ Hay PJ. Acupuncture for depression. Cochrane Database Syst Rev. (2018) 3:CD004046. 10.1002/14651858.CD004046.pub4 29502347 PMC6494180

[19] Hung CY Wu XY Chung VC Tang EC Wu JC Lau AY. Overview of systematic reviews with meta-analyses on acupuncture in post-stroke cognitive impairment and depression management. Integr Med Res. (2019) 8:145–59. 10.1016/j.imr.2019.05.001 31304087 PMC6600770

[20] Wen X Li K Wen H Wang Q Wu Z Yao X . Acupuncture-related therapies for Parkinson's disease: a meta-analysis and qualitative review. Front Aging Neurosci. (2021) 13:676827. 10.3389/fnagi.2021.676827 34276340 PMC8282198

[21] Vataja R Pohjasvaara T Mantyla R Ylikoski R Leskela M Kalska H . Depression-executive dysfunction syndrome in stroke patients. Am J Geriatr Psychiatry. (2005) 13:99–107. 10.1097/00019442-200502000-00003 15703318

[22] Pohjasvaara T Leskela M Vataja R Kalska H Ylikoski R Hietanen M . Post-stroke depression, executive dysfunction and functional outcome. Eur J Neurol. (2002) 9:269–75. 10.1046/j.1468-1331.2002.00396.x 11985635

[23] Jaywant A DelPonte L Kanellopoulos D O'Dell MW Gunning FM. The structural and functional neuroanatomy of post-stroke depression and executive dysfunction: a review of neuroimaging findings and implications for treatment. J Geriatr Psychiatry Neurol. (2022) 35:3–11. 10.1177/0891988720968270 33073704

[24] Respino M Hoptman MJ Victoria LW Alexopoulos GS Solomonov N Stein AT . Cognitive control network homogeneity and executive functions in late-life depression. Biol Psychiatry Cogn Neurosci Neuroimaging. (2020) 5:213–21. 10.1016/j.bpsc.2019.10.013 31901436 PMC7010539

[25] Roussel M Dujardin K Henon H Godefroy O. Is the frontal dysexecutive syndrome due to a working memory deficit? Evidence from patients with stroke. Brain. (2012) 135 (Pt 7):2192–201. 10.1093/brain/aws132 22637543

[26] Kuceyeski A Navi BB Kamel H Relkin N Villanueva M Raj A . Exploring the brain's structural connectome: A quantitative stroke lesion-dysfunction mapping study. Hum Brain Mapp. (2015) 36:2147–60. 10.1002/hbm.22761 25655204 PMC4414746

[27] Foulon C Cerliani L Kinkingnehun S Levy R Rosso C Urbanski M . Advanced lesion symptom mapping analyses and implementation as BCBtoolkit. Gigascience. (2018) 7:1–17. 10.1093/gigascience/giy004 29432527 PMC5863218

[28] Seeley WW Crawford RK Zhou J Miller BL Greicius MD. Neurodegenerative diseases target large-scale human brain networks. Neuron. (2009) 62:42–52. 10.1016/j.neuron.2009.03.024 19376066 PMC2691647

[29] Liu Y Hu G Yu Y Jiang Z Yang K Hu X . Structural and functional reorganization within cognitive control network associated with protection of executive function in patients with unilateral frontal gliomas. Front Oncol. (2020) 10:794. 10.3389/fonc.2020.00794 32528887 PMC7266965

[30] Seeley WW Menon V Schatzberg AF Keller J Glover GH Kenna H . Dissociable intrinsic connectivity networks for salience processing and executive control. J Neurosci. (2007) 27:2349–56. 10.1523/JNEUROSCI.5587-06.2007 17329432 PMC2680293

[31] Sakai K Passingham RE. Prefrontal set activity predicts rule-specific neural processing during subsequent cognitive performance. J Neurosci. (2006) 26:1211–8. 10.1523/JNEUROSCI.3887-05.2006 16436608 PMC6674561

[32] Tops M Boksem MA. A potential role of the inferior frontal gyrus and anterior insula in cognitive control, brain rhythms, and event-related potentials. Front Psychol. (2011) 2:330. 10.3389/fpsyg.2011.00330 22084637 PMC3212750

[33] Egorova N Cumming T Shirbin C Veldsman M Werden E Brodtmann A. Lower cognitive control network connectivity in stroke participants with depressive features. Transl Psychiatry. (2018) 7:4. 10.1038/s41398-017-0038-x 29520018 PMC5843603

[34] Liston C Chen AC Zebley BD Drysdale AT Gordon R Leuchter B . Default mode network mechanisms of transcranial magnetic stimulation in depression. Biol Psychiatry. (2014) 76:517–26. 10.1016/j.biopsych.2014.01.023 24629537 PMC4209727

[35] Alexopoulos GS Hoptman MJ Kanellopoulos D Murphy CF Lim KO Gunning FM. Functional connectivity in the cognitive control network and the default mode network in late-life depression. J Affect Disord. (2012) 139:56–65. 10.1016/j.jad.2011.12.002 22425432 PMC3340472

[36] Ye M Yang T Qing P Lei X Qiu J Liu G. Changes of functional brain networks in major depressive disorder: a graph theoretical analysis of resting-state fMRI. PLoS ONE. (2015) 10:e0133775. 10.1371/journal.pone.0133775 26327292 PMC4556670

[37] Tan TT Wang D Huang JK Zhou XM Yuan X Liang JP . Modulatory effects of acupuncture on brain networks in mild cognitive impairment patients. Neural Regen Res. (2017) 12:250–8. 10.4103/1673-5374.200808 28400807 PMC5361509

[38] Xiong J Zhang Z Ma Y Li Z Zhou F Qiao N . The effect of combined scalp acupuncture and cognitive training in patients with stroke on cognitive and motor functions. NeuroRehabilitation. (2020) 46:75–82. 10.3233/NRE-192942 32039871

[39] Hori E Takamoto K Urakawa S Ono T Nishijo H. Effects of acupuncture on the brain hemodynamics. Auton Neurosci. (2010) 157:74–80. 10.1016/j.autneu.2010.06.007 20605114

[40] Liu G Ma HJ Hu PP Tian YH Hu S Fan J . Effects of painful stimulation and acupuncture on attention networks in healthy subjects. Behav Brain Funct. (2013) 9:23. 10.1186/1744-9081-9-23 23758880 PMC3680197

[41] American Psychiatric Association. DSM-5. 5th ed. Arlington, VA: American Psychiatric Association (2013).

[42] World Health Organization. A Proposed Standard International Acupuncture Nomenclature: Report of a WHO Scientific Group. Geneva (1991). Available online at: https://apps.who.int/iris/bitstream/handle/10665/40001/9241544171_eng.pdf?sequence=1

[43] Wang D. The Third WHO regional workshop on the standardization of acupuncture nomenclature. J Trad Chin Med. (1988) 8:221. 3216669

[44] World Health Organization. WHO Standard Acupuncture Point Locations in the Western Pacific Region. Geneva: World Health Organization (2008).

[45] Liu L Chen W Zhou H Duan W Li S Huo X . Chinese Stroke Association guidelines for clinical management of cerebrovascular disorders: executive summary and 2019 update of clinical management of ischaemic cerebrovascular diseases. Stroke Vasc Neurol. (2020) 5:159–76. 10.1136/svn-2020-000378 32561535 PMC7337371

[46] Qiu K Jing M Sun R Yang J Liu X He Z . The status of the quality control in acupuncture-neuroimaging studies. Evid Based Comp Altern Med. (2016) 2016:3685785. 10.1155/2016/3685785 27242911 PMC4875991

[47] Hamilton M. A rating scale for depression. J Neurol Neurosurg Psychiatry. (1960) 23:56–62. 10.1136/jnnp.23.1.56 14399272 PMC495331

[48] Hamilton M. Development of a rating scale for primary depressive illness. Br J Soc Clin Psychol. (1967) 6:278–96. 10.1111/j.2044-8260.1967.tb00530.x 6080235

[49] Leucht S Fennema H Engel R Kaspers-Janssen M Lepping P Szegedi A. What does the HAMD mean? J Affect Disord. (2013) 148:243–8. 10.1016/j.jad.2012.12.001 23357658

[50] Hautzinger M. [The Beck Depression Inventory in clinical practice]. Nervenarzt. (1991) 62:689–96. 1770969

[51] Richter P Werner J Heerlein A Kraus A Sauer H. On the validity of the beck depression inventory. A review. Psychopathology. (1998) 31:160–8. 10.1159/000066239 9636945

[52] Scarpina F Tagini S. The stroop color and word test. Front Psychol. (2017) 8:557. 10.3389/fpsyg.2017.00557 28446889 PMC5388755

[53] Giovagnoli AR Del Pesce M Mascheroni S Simoncelli M Laiacona M Capitani E. Trail making test: normative values from 287 normal adult controls. Ital J Neurol Sci. (1996) 17:305–9. 10.1007/BF01997792 8915764

[54] Sanchez-Cubillo I Perianez JA Adrover-Roig D Rodriguez-Sanchez JM Rios-Lago M Tirapu J . Construct validity of the Trail Making Test: role of task-switching, working memory, inhibition/interference control, and visuomotor abilities. J Int Neuropsychol Soc. (2009) 15:438–50. 10.1017/S1355617709090626 19402930

[55] Wechsler D. Wechsler Adult Intelligence Scale. 3rd ed. San Antonio, TX: The Psychological Corporation (1997). 10.1007/978-1-4419-1698-3_101553

[56] Bright P Hale E Gooch VJ Myhill T van der Linde I. The National Adult Reading Test: restandardisation against the Wechsler Adult Intelligence Scale-Fourth edition. Neuropsychol Rehabil. (2018) 28:1019–27. 10.1080/09602011.2016.1231121 27624393

[57] Zhang N Du S Tang Z Zheng M Ma G. Effect of water supplementation on cognitive performances and mood among male college students in Cangzhou, China: study protocol of a randomized controlled trial. Int J Environ Res Public Health. (2017) 14:966. 10.3390/ijerph14090966 32962317 PMC5615503

[58] Chiaravalloti ND Weber E Wylie G Dyson-Hudson T Wecht JM. Patterns of cognitive deficits in persons with spinal cord injury as compared with both age-matched and older individuals without spinal cord injury. J Spinal Cord Med. (2020) 43:88–97. 10.1080/10790268.2018.1543103 30508409 PMC7006756

[59] Wechsler D. Wechsler Abbreviated Scale of Intelligence Manual (WASI). San Antonio, TX: Harcourt Assessment (1999).

[60] Maldjian JA Laurienti PJ Kraft RA Burdette JH. An automated method for neuroanatomic and cytoarchitectonic atlas-based interrogation of fMRI data sets. Neuroimage. (2003) 19:1233–9. 10.1016/S1053-8119(03)00169-1 12880848

[61] Zhang JS. [Effect of shugan jiannao tiaoyu tablets (SJTT) on hypothalamic corticotrophin releasing hormone gene expression in model rat of post-stroke depression]. Zhongguo Zhong Yao Za Zhi. (2008) 33:2037–40. 19086649

[62] Sun PY Li PF Wang T Wu J Li N Liu H . [Effect of Tongdu Tiaoshen acupuncture on PI3K/Akt/mTOR signaling pathway and autophagy-related proteins of hippocampus in rats with post-stroke depression]. Zhongguo Zhen Jiu. (2020) 40:1205–10. 10.13703/j.0255-2930.20200522-k0006 33788489

[63] De Raedt R Koster EH. Understanding vulnerability for depression from a cognitive neuroscience perspective: a reappraisal of attentional factors and a new conceptual framework. Cogn Affect Behav Neurosci. (2010) 10:50–70. 10.3758/CABN.10.1.50 20233955

[64] Disner SG Beevers CG Haigh EA Beck AT. Neural mechanisms of the cognitive model of depression. Nat Rev Neurosci. (2011) 12:467–77. 10.1038/nrn3027 21731066

